# Changes in the amino acid profiles and free radical scavenging activities of *Tenebrio molitor* larvae following enzymatic hydrolysis

**DOI:** 10.1371/journal.pone.0196218

**Published:** 2018-05-04

**Authors:** Yujiao Tang, Trishna Debnath, Eun-Ju Choi, Young Wook Kim, Jung Pyo Ryu, Sejin Jang, Sang Uk Chung, Young-Jin Choi, Eun-Kyung Kim

**Affiliations:** 1 School of Bio-science and Food Engineering, Changchun University of Science and Technology, Changchun, China; 2 Division of Food Bioscience, College of Biomedical and Health Sciences, Konkuk University, Chungju, Republic of Korea; 3 Department of Physical Education, College of Education, Daegu Catholic University, Gyeongsan, Republic of Korea; 4 R&D Center, Korean Edible Insect Laboratory Knowledge Cooperative Association, Seoul, Republic of Korea; Jadavpur University, INDIA

## Abstract

*Tenebrio molitor* (*T*. *molitor*) larvae provide food at low environmental cost and contribute positively to livelihoods. In this research, we compared the amino acids compositions and antioxidant activities of various extracts of *T*. *molitor* to enhance their quality as food. For the comparison, distilled water extracts, enzymatic hydrolysates, and condensed enzymatic hydrolysates of *T*. *molitor* larvae were prepared. Their amino acids (AAs) profiles and antioxidant activities, including ferric-reducing antioxidant power, oxygen radical absorption capacity, and DPPH, hydroxyl radical, and hydrogen peroxide radical scavenging properties assay were analyzed. DW extracts had the lowest AAs contents and antioxidant activity compared with enzymatic extracts. Condensed hydrolysates with a combination of alcalase and flavourzyme (C-A+F) exhibited the highest levels of total free AAs (11.1759 g/100 g). C-A+F produced higher total hydrolyzed AAs (32.5292 g/100 g) compared with the other groups. The C-A+F possessed the strongest antioxidant activity. Notably, the antioxidant activities of the hydrolysates and the total hydrolyzed AAs amount were correlated. Taken together, our findings showed that C-A+F was a promising technique for obtaining extracts of *T*. *molitor* larvae with antioxidant activity as potential nutritious functional food.

## Introduction

Edible insects have played an important role in the history of human nutrition and are traditionally consumed in many parts of the world [1]. Moreover; edible insects are used to supplement the diets of approximately 2 billion people and are attracting increased interest in the human nutrition [2,3]. Insects are also attracting attention owing to their high protein content (Table 1) and bioactive peptides and about 2,000 edible insect species have been identified to date [4–7]. Moreover, insects are considered acceptable as food products because of their nutritive value and taste. Now a days Consequently, they may represent an important food source in the future [3]. Meanwhile, *Tenebrio molitor* (*T*. *molitor*) larvae, also known as yellow mealworms, belong to the family of Tenebrionidae and are generally considered pests because they feed on stored grains. However, these larvae are edible, and baked or fried *T*. *molitor* are available commercially as a healthful snack food, and one of the most prominent edible insects across the globe [7].

**Table 1:** Average protein contents of prominent insects.

Common names (scientific name)	Protein contents (g/100 g fresh weight)
**Yellow mealworm (*Tenebrio molitor*)**	25
**Crickets (*Acheta domesticus*)**	23
**Termites (*Macrotermes nigeriensis*)**	22
**Palmworm beetles (*Rhynchophorus ferrugineus*)**	20
**Locusts (*Locusta migratoria*)**	17
**Silkworm (*Bombyx mori*)**	15
**Grasshoppers (*Melanoplus femurrubrum*)**	14

Hydrolysis with enzymes has been used for centuries to improve the taste and quality of food products. There are many applications of enzymes in the food industry, including flavor enhancers and for fish and seafood processing [8]. However, few studies have examined the effects of enzymatic hydrolysis of edible insects for applications in the food industry.

The protein rich *Tenebrio molitor* Larvae abounds amino acid such as isoleucine, leucine, lysine and contains fatty acid with high component of oleic acid, linoleic acid, palmitic acid that has significance in human diet. *Tenebrio molitor* can be considered as an alternative protein source in the human diet [9]. Enzymatic hydrolysis is an established method for enhancing the functional characteristics of proteins by modifying their solubility, viscosity, and emulsifying and foaming properties, thereby improving their industrial utility. During enzymatic hydrolysis, proteins are cleaved to smaller peptides and free amino acids (FAAs). FAAs and low-molecular-weight peptides (hydrolysates) are currently being used in various applications, including as protein-rich ingredients [10,11]. Recently alcalase and flavourzyme have been used as effective enzymes for the hydrolysis of proteins [12,13]. Kristinsson and Rasco (2000) reported that alcalase can be used in the hydrolysis of proteins because it provides rapid, efficient hydrolysis [14]. Additionally, flavourzyme is important for biocatalysis because of the selectivity, controllability, and efficiency of its catalytic function. Flavourzyme has been studied for its unique structural and mechanistic activities and is attracting attention in industrial biocatalytic applications [15]. In addition, many peptides present in protein hydrolysates possess strong biological activities [16–18]. Several studies have reported that peptides from legumes, chickpeas, corn, soybeans, eggs, milk, and various aquatic products (e.g., fish and shrimps) are good sources of antioxidants, which can be extracted via enzymatic hydrolysis [4,19–23].

To date, however, no reports have provided a comparison of amino acids (AAs) profiles of distilled water (DW) extracts and enzymatic hydrolysis of *T*. *molitor* larvae or described the free radical scavenging activities of proteins from *T*. *molitor* larvae.

Therefore, in the present study, we performed DW extraction and enzymatic hydrolysis of *T*. *molitor* larvae with alcalase and flavourzyme and compared the AAs compositions and free radical scavenging activities among extracts.

## Materials and methods

### Materials

Third instar *T*. *molitor* larvae were purchased from Cricket Farm (Hwaseong-si, Gyeonggi-do, Korea). 1,1-Diphenyl-2-picrylhydrazyl (DPPH), alcalase, and flavourzyme were obtained from Sigma-Aldrich (St. Louis, MO, USA). The other chemicals and reagents used in this study were of analytical grade and were commercially available.

### Preparation of *T*. *molitor* larvae samples

*T*. *molitor* larvae were starved for 48 h to empty the intestines, followed by washing with distilled water (DW) and drying under mid-infrared light. After the moisture content reached to 4%, *T*. *molitor* larvae were pressed to remove oils for 15 min (YJ-319; Youngjin Machine Co. Ltd., Seoul, Korea). The resulting larvae were ground with a blender (Quiet One; Vitamix, USA) and sieved through a 100-mesh-size sieve. *T*. *molitor* larvae (10 g) were then subjected to a 2-h extraction in 100 mL DW. The extract was filtered (0.25 μm) and lyophilized in a freeze dryer for 5 days. Enzymatic hydrolysates were obtained according to the method described by Park et al. (2005) [24]. Briefly, 100 mL DW was added to 10 g of powder sample, and 10 μL of each enzyme was then added after pre-incubation for 30 min at 55°C. The enzymatic hydrolysis reactions were performed for 8 h at 55°C and pH 7 to achieve an optimum hydrolytic level and immediately heated to inactivate the enzyme at 100°C for 10 min. For preparation of the condensed hydrolysates, enzymatic extracts were concentrated by evaporation to 50% brix. The mixture was then rapidly cooled to 20–25°C in an ice bath. The lysates were lyophilized and then stored at -20°C until use.

### Analysis of approximate compositions

The *T*. *molitor* larvae samples were analyzed for dry matter (DM), crude protein (CP), ether extract (EE), crude protein (CF) and carbohydrate according to the AOAC. Briefly, DM was determined by drying a portion (2 g) from the composite sample for each year to constant weight in an oven at 105°C. Ash was determined by incinerating the dried sample (2 g) in a muffle furnace at 600°C for 6 h. CP was estimated by the macro-Kjeldahl method, and calculated by multiplying the measured nitrogen by 6.25 (method 978.04, AOAC, 1990). An aliquot of 3 g was used to determine CF by extracting with petroleum ether (40–60°C) in a Soxhlet apparatus. The CF was determined by alternately digesting the dried, defatted sample (2 g) in 1.25% HCl and 1.25% NaOH (method 930.10, AOAC, 1990). Carbohydrate levels were calculated by subtracting the total sum of crude protein, crude fat, and crude fibre from 100% dry weight sample [25].

### AAs analysis

**Fig 1:**
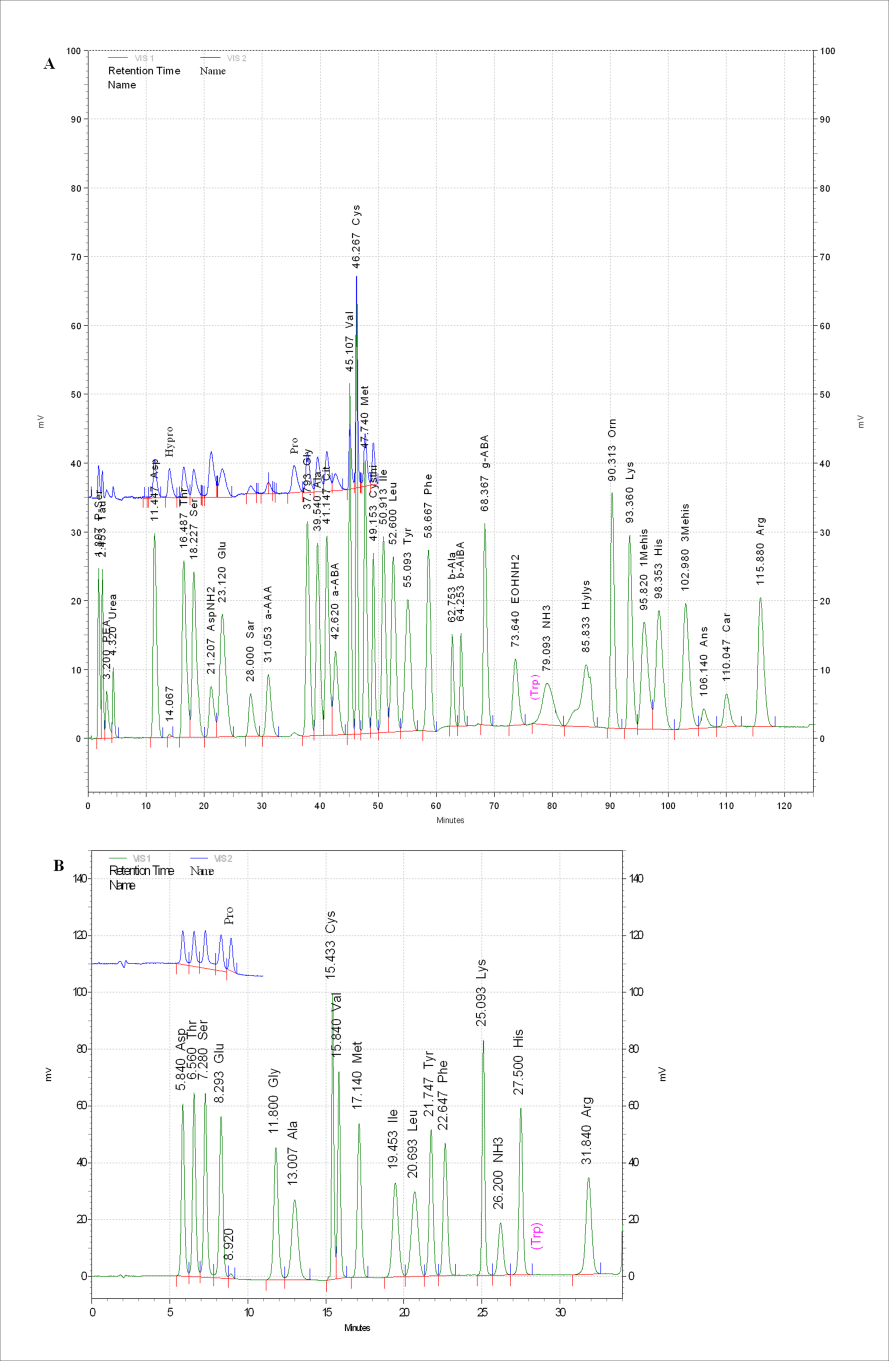
Standards AAs peaks. A, FAAs standards peaks; B, HAAs standards peaks. AAs, amino acids; FAAs, free amino acids; HAAs, hydrolyzed amino acids.

For analysis of FAAs, the samples were hydrolyzed in 5% trichloroacetic acid (TCA), and for the analysis of hydrolyzed amino acids (HAAs), the samples were hydrolyzed in 6 N hydrochloric acid in vacuum-sealed tubes at 110°C for 24 h. The AAs content of the various hydrolysates was determined using an AAs analyzer (HITACHI L-8900 Amino Acid Analyzer; Hitachi High-Technologies Corporation, Tokyo, Japan) with postcolumn derivatization using ninhydrin. The column (60 mm × 4.6 mm) was packed with 3-μm particles and equipped with a 40 mm × 4.6 mm guard column (ammonia filter). The AAs standard solution (L-8500; Wako Chemical, Osaka, Japan) was used for identification and quantification of FAAs by measuring the absorption of reaction products with ninhydrin at 570 and 440 nm. The standards AAs peaks were shown in Fig 1. The AAs were expressed as g/100 g.

### Analysis of free radical scavenging activities

#### DPPH radical scavenging activity

The DPPH scavenging activity of various extracts was measured according to the methods of Blois (1958), with slight modifications. The DPPH solution (1.5 × 10^−4^ M, 100 μL) was mixed with or without each extract (100 μL), and the mixture was then incubated at room temperature for 30 min [26]. After standing for 30 min, absorbance was recorded at 540 nm using a microplate reader, and the percentage of scavenging activity was calculated using the following equation:

$$Inhibition\;\left(\%\right)=(A_{control}-A_{sample})\;/\;A_{control}\times 100$$

where A_control_ is the absorbance of the reaction mixture without sample, and A_sample_ is the absorbance of the reaction mixture with sample at 540 nm.

Standard calibration curves were constructed by plotting the percent inhibition against that of trolox. The ratio between the percent inhibition of the sample and the gradient of the trolox calibration curve (x = [y–b] / a) was determined as the trolox equivalent antioxidant capacity (TEAC) and expressed in μM trolox equivalents (TE/mg). The TEAC values of the samples were calculated using the following equation:

TEAC(μMTE/mg)=(Inhibition[%]–b)/a

#### Hydroxyl radical scavenging activity

Hydroxyl radical scavenging activity of various extracts was determined according to the method of Chung et al (1997) [27]. The hydroxyl radical was generated by the Fenton reaction in the presence of FeSO_4_. A reaction mixture containing 0.1 mL of 10 mM FeSO_4_, 10 mM EDTA, and 10 mM 2-deoxyribose was mixed with 0.1 mL of the extract solution, after which 0.1 M phosphate buffer (pH 7.4) was added until the total volume reached 0.9 mL. Subsequently, 0.1 mL of 10 mM H_2_O_2_ was added to the reaction mixture, and the samples were incubated at 37°C for 4 h. After incubation, 0.5 mL of 2.8% TCA and 0.5 mL of 1.0% tert-butyl alcohol (TBA) were added to the mixture, and the mixture was placed in a boiling water bath for 10 min. Absorbance was measured at 532 nm, and the percent scavenging activity was calculated using the following equation:

$$Inhibition\;\left(\%\right)=(A_{control}-A_{sample})\;/\;A_{control}\times 100$$

where A_control_ is the absorbance of the reaction mixture without the sample, and A_sample_ is the absorbance of the reaction mixture with the sample at 532 nm.

Standard calibration curves were constructed and TEAC values were determined as described in section DPPH.

#### Ferric reducing antioxidant power (FRAP)

FRAP assays were carried out according to the methods of Benzie and Strain (1996) [28]. Fresh working solution was prepared by mixing acetate buffer, 2,4,6-tri(2-pyridyl)-s-triazine (TPTZ) solution, and FeCl_3_•6H_2_O solution and then warmed to 37°C before use. Each extract was allowed to react with FRAP solution in a dark room at room temperature for 30 min. Readings of the colored product were then taken at 595 nm, and the percent scavenging activity was calculated using the following equation:

TEAC(μMTE/mg)=(Sample–b)/a

where A_control_ is the absorbance of the reaction mixture without the sample, and A_sample_ is the absorbance of the reaction mixture with the sample at 595 nm.

#### Oxygen radical absorbance capacity (ORAC)

For ORAC assays, the method of Ou et al (2001) was used with some slight modifications [29]. A working solution of FL and AAPH radical were prepared daily. Samples, blanks, and standards were placed in a 96-microwell plate, and AAPH was added. The fluorescence was then measured immediately after the addition of AAPH, and measurements were taken every 5 min.

$$ORAC\;(\mu M\;TE)=(C_{Trolox}\times (AUC_{sample}-AUC_{blank})\times k)\;/\;(AUC_{sample}-AUC_{blank})$$

where C_Trolox_ is the concentration (lM) of trolox (20 lM), k is the sample dilution factor, and the area under the curve (AUC) is the area below the fluorescence decay curve of the sample, blank, and trolox, calculated by applying the following formula:

AUC=(0.5+f1/f0+f2/f0+…+fn/f0)×5

where f0 is the initial fluorescence, and fn is the fluorescence at time n.

### Statistical analysis

The experiments shown are the mean ± standard error (SE) of data from at least three experiments. Statistical analyses were performed using SAS statistical software (SAS Institute, Cary, NC, USA). Treatment effects were analyzed using one-way analysis of variance, followed by Dunnett’s multiple range tests. Differences with *p* values of less than 0.05 were considered significant.

## Results

### Approximate compositions and yields

In the present study, DW extraction, enzymatic hydrolysis, and condensed enzymatic hydrolysis with alcalase, flavourzyme, or both alcalase and flavourzyme were carried out, yielding seven samples: DW extracts (DW), enzymatic hydrolysates with alcalase (A), condensed enzymatic hydrolysates with alcalase (C-A), enzymatic hydrolysates with flavourzyme (F), condensed enzymatic hydrolysates with flavourzyme (C-F), enzymatic hydrolysates with alcalase and flavourzyme (A+F), and condensed enzymatic hydrolysates with alcalase and flavourzyme (C-A+F). The yields ranged from 10.0% to 38.7% (Table 2). The highest yield was found from alcalase hydrolysis, whereas DW extraction resulted in the lowest total yield. Meanwhile, the crud protein contents ranged from 4.01% to 39.41% (Table 2); C-A+F (39.41%) and C-A (34.45%) showed higher crud protein contents than the other samples. The content of EE was the highest in the C-A, and the CF was the highest in C-A+F.

**Table 2:** Yield and aproximate composition of *Tenebrio molitor* larvae following enzymatic hydrolysis.

Sample	Yield%	DM%	CP%	EE%	CF%	Ash%	CHO%
**DW**	10	1.24	4.01	0.97	0.87	2.86	90.05
**A**	38.7	1.08	8.59	1.87	1.58	2.74	84.14
**C-A**	19.8	1.58	34.45	3.21	7.84	2.46	50.46
**F**	14	1.74	6.26	2.21	1.67	2.28	85.84
**C-F**	8.2	1.42	18.6	1.42	2.43	2.54	73.59
**A+F**	36.4	1.51	13.07	1.39	1.63	2.36	80.04
**C-A+F**	16.7	1.37	39.41	3.4	8.33	2.43	45.06

DM, dry matter; CP, crude protein; EE, ether extract; CF, crude fibre; CHO, carbohydrate; DW, distilled water extract; A, alcalase hydrolysis; C-A, condensed alcalase hydrolysis; F, flavourzyme hydrolysis; C-F, condensed flavourzyme hydrolysis; A+F, alcalase+flavourzyme hydrolysis; C-A+F, condensed alcalase+flavourzyme hydrolys

### Amino acid composition

The FAAs contents of the samples are presented in Table 3; we detected 18 AAs in the samples, including aspartic acid (Asp), glutamic acid (Glu), serine (Ser), glycine (Gly), histidine (His), arginine (Arg), threonine (Thr), alanine (Ala), proline (Pro), tyrosine (Tyr), valine (Val), methionine (Met), isoleucine (Ile), leucine (Leu), and phenylalanine (Phe). Here, significant variations in FAAs contents among the different samples were observed. DW extracted a lower amount of total FAAs compared with enzymatic extraction. An extracted lower total FAAs compared with F (Table 3). Furthermore, A+F (3.8607 g/100 g) and C-A+F (11.1187 g/100 g) extracted the most abundant levels of total FAAs (Table 3).

**Table 3:** Free amino acids (FAAs) composition of *T*. *molitor* larvae following enzymatic hydrolysis (g/100 g).

	Sample						
FAAs	DW	A	C-A	F	C-F	A+F	C-A+F
	AA/FAA	AA/FAA	AA/FAA	AA/FAA	AA/FAA	AA/FAA	AA/FAA
Asp	0.0029	0.0103	0.0462	0.0381	0.1173	0.1484	0.2991
	0.5392%	1.6099%	1.4377%	3.0955%	3.1452%	3.8439%	2.6763%
Thr	0.0129	0.014	0.0566	0.0486	0.1479	0.2111	0.5132
	2.3987%	2.1882%	1.7613%	3.9487%	3.9657%	5.4679%	4.5920%
Ser	0	0.0194	0.1004	0.0494	0.1489	0.2414	0.6047
	0.0000%	3.0322%	3.1243%	4.0136%	3.9925%	6.2528%	5.4107%
Asn	0.0012	0.0073	0.0452	0.032	0.1046	0.1629	0.3904
	0.2231%	1.1410%	1.4066%	2.5999%	2.8047%	4.2194%	3.4932%
Glu	0.026	0.0205	0.1038	0.0562	0.1799	0.1518	0.4535
	4.8345%	3.2041%	3.2301%	4.5661%	4.8237%	3.9319%	4.0578%
Pro	0.0779	0.1667	0.8684	0.2163	0.6534	0.301	0.8541
	14.4849%	26.0550%	27.0235%	17.5739%	17.5198%	7.7965%	7.6423%
Gly	0.0121	0.0093	0.0556	0.0233	0.0663	0.098	0.2885
	2.2499%	1.4536%	1.7302%	1.8931%	1.7777%	2.5384%	2.5814%
Ala	0.0816	0.0565	0.4113	0.0989	0.2954	0.361	0.9495
	15.1729%	8.8309%	12.7991%	8.0354%	7.9206%	9.3506%	8.4960%
Val	0.0554	0.045	0.1997	0.0875	0.2644	0.3682	0.9171
	10.3012%	7.0334%	6.2144%	7.1092%	7.0894%	9.5371%	8.2061%
Ile	0.0193	0.028	0.1299	0.0563	0.1741	0.2313	0.6777
	3.5887%	4.3764%	4.0423%	4.5743%	4.6682%	5.9911%	6.0639%
Leu	0.0214	0.0401	0.1569	0.0966	0.2992	0.4867	1.4019
	3.9792%	6.2676%	4.8825%	7.8486%	8.0225%	12.6065%	12.5440%
Tyr	0.0573	0.0513	0.2688	0.0864	0.2565	0.2555	1.0961
	10.6545%	8.0181%	8.3647%	7.0198%	6.8776%	6.6180%	9.8077%
Phe	0.0133	0.0363	0.1081	0.0463	0.1406	0.2477	0.7337
	2.4730%	5.6736%	3.3639%	3.7618%	3.7699%	6.4159%	6.5650%
Lys	0.021	0.0218	0.1118	0.0884	0.2637	0.3172	0.9521
	3.9048%	3.4073%	3.4791%	7.1823%	7.0707%	8.2161%	8.5192%
His	0.0546	0.0218	0.1419	0.0571	0.17	0.1692	0.5027
	10.1525%	3.4073%	4.4157%	4.6393%	4.5583%	4.3826%	4.4981%
Arg	0.0779	0.0712	0.3506	0.1361	0.4065	0.0052	0.0252
	14.4849%	11.1285%	10.9102%	11.0578%	10.8996%	0.1347%	0.2255%
Cys	0	0.0135	0.0369	0	0	0.0267	0.319
	0.0000%	2.1100%	1.1483%	0.0000%	0.0000%	0.6916%	2.8544%
Met	0.0029	0.0065	0.0214	0.0132	0.041	0.0773	0.1974
	0.5392%	1.0159%	0.6659%	1.0725%	1.0993%	2.0022%	1.7663%
Total	0.5378	0.6398	3.2135	1.2308	3.7295	3.8607	11.1759

DW, distilled water extract; A, alcalase hydrolysis; C-A, condensed alcalase hydrolysis; F, flavourzyme hydrolysis; C-F, condensed flavourzyme hydrolysis; A+F, alcalase+flavourzyme hydrolysis; C-A+F, condensed alcalase+flavourzyme hydroly

The HAAs compositions of the samples examined in this study are shown in Table 4. The results showed that C-A and C-A+F produced higher levels of HAAs than the other procedures. In contrast, DW produced the lowest total levels of HAAs among the samples.

**Table 4:** Hydrolyzed amino acids (HAAs) composition of *Tenebrio molitor* larvae following enzymatic hydrolysis (g/100 g).

	Sample						
HAAs	DW*	A	C-A	F	C-F	A+F	C-A+F
Asp	0.1599	0.7695	3.1024	0.5199	1.5442	1.1479	3.1536
Thr	0.0830	0.3647	1.4683	0.2376	0.6939	0.5478	1.5137
Ser	0.0754	0.4203	1.6322	0.2605	0.7682	0.5841	1.6517
Glu	0.3404	1.1152	4.4645	0.8295	2.4584	1.6534	4.6911
Pro	0.3842	0.5349	2.3569	0.4021	1.2139	0.8667	2.3354
Gly	0.1175	0.4131	1.6037	0.2532	0.7542	0.6169	1.6339
Ala	0.1527	0.5757	2.3932	0.2987	0.8970	0.9104	2.4603
Val	0.1137	0.4278	1.7964	0.2740	0.8318	0.7606	1.8779
Ile	0.0642	0.2887	1.2275	0.1975	0.5912	0.5313	1.3451
Leu	0.0987	0.6023	2.4214	0.3822	1.1255	0.9518	2.5298
Tyr	0.0804	0.6051	2.3434	0.3784	1.0747	0.5188	2.1687
Phe	0.0515	0.3133	1.2573	0.2147	0.6186	0.4875	1.3362
Lys	0.1063	0.4932	2.0405	0.3493	1.0272	0.7724	2.0928
His	0.0942	0.2548	1.0389	0.1689	0.5068	0.3911	1.0618
Arg	0.0674	0.4593	1.9251	0.3172	0.9604	0.3354	1.5779
Cys	0.0354	0.1166	0.4170	0.0830	0.2370	0.1626	0.4674
Met	0.0170	0.1846	0.7336	0.0918	0.3027	0.2010	0.6319
Total	2.0419	7.9391	32.2222	5.2584	15.6058	11.4398	32.5292

*DW, distilled water extract; A, alcalase hydrolysis; C-A, condensed alcalase hydrolysis; F, flavourzyme hydrolysis; C-F, condensed flavourzyme hydrolysis; A+F, alcalase+flavourzyme hydrolysis; C-A+F, condensed alcalase+flavourzyme hydrolysis

### Antioxidant activities

**Fig 2:**
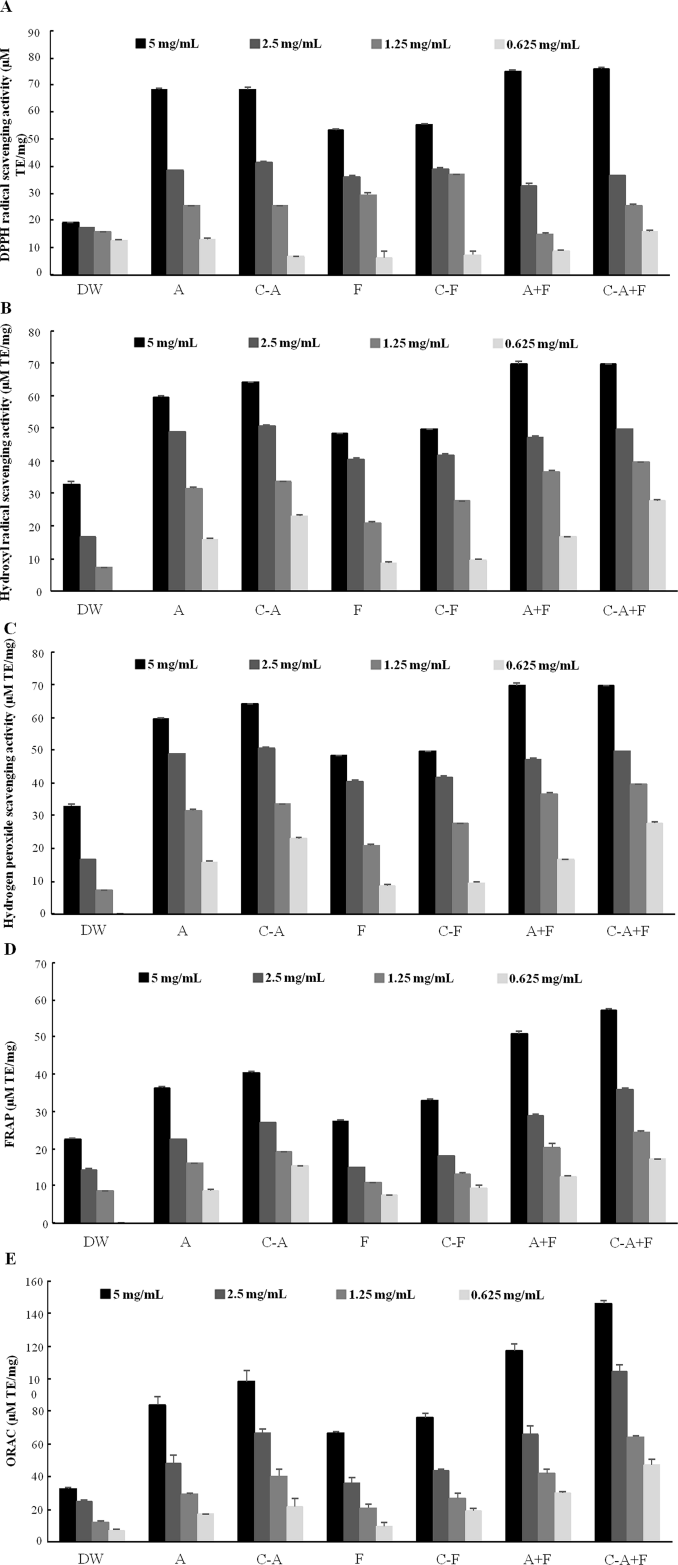
Various antioxidant activities of *Tenebrio molitor* larvae. A,1,1-Diphenyl-2-picryl-hydrazyl (DPPH) radical scavenging activity; B, hydroxyl radial scavenging activity; C, hydrogen peroxide scavenging activities; D, oxygen radical absorbance capacity (ORAC); E, ferric reducing antioxidant power (FRAP). Data are presented as the mean ± SD of triplicate determinations. DW, distilled water extract; A, alcalase hydrolysis; C-A, condensed alcalase hydrolysis; F, flavourzyme hydrolysis; C-F, condensed flavourzyme hydrolysis; A+F, alcalase+flavourzyme hydrolysis; C-A+F, condensed alcalase+flavourzyme hydrolysis.

All samples scavenged free radicals in a concentration-dependent manner (Fig 2). The IC_50_ values of ORAC, FRAP, and DPPH, hydroxyl radical, and hydrogen peroxide scavenging activities are shown in Table 5. DW showed the lowest antioxidant activity of all the samples, whereas A+F and C-A+F exhibited the highest antioxidant activity. Moreover, A and C-A showed higher scavenging activities than F and C-F. However, there were no significant differences in antioxidant activities between A and C-A or between F and C-F (Table 5, Fig 2). These results were positively correlated with the HAAs composition. We also observed similar inhibitory activities for both A+F and C-A+F when used at a concentration of 5 mg/mL. A+F showed the highest levels of DPPH, hydroxyl free radical, and hydrogen peroxide scavenging activities at 83, 38, and 73 μM TE/mg, respectively, whereas C-A+F showed scavenging activities of 83, 40, and 73 μM TE/mg, respectively (Fig 2A–2C). Additionally, C-A+F showed higher FRAP and ORAC activities than A+F when used at a concentration of 5 mg/mL (Fig 2D and 2E).

**Table 5:** IC_50_ values of various antioxidant activities of *Tenebrio molitor* larvae following enzymatic hydrolysis.

	IC_50_* (μM TE/mg)				
	DPPH	Hydroxyl	Hydrogen peroxide	FRAP	ORAC
DW**	46.12 ± 0.3***^,a^	17.06 ± 0.1^a^	10.16 ± 0.1^a^	11.56 ± 0.2^a^	7.706±0.1^a^
A	5.32 ± 0.1^c^	8.81 ± 0.5^c^	6.41 ± 0.5^c^	7.22 ± 0.4^c^	2.709±0.5^c^
C-A	5.14 ± 0.2^c^	8.48 ± 0.5^c^	6.06 ± 1.0^c^	6.65 ± 0.6^c^	1.950±0.4^c^
F	6.17 ± 0.1^b^	16.26 ± 0.8^b^	7.77 ± 2.0^b^	10.04 ± 0.5^b^	6.662±0.6^b^
C-F	6.58 ± 0.2^b^	15.19 ± 1.0^b^	7.74 ± 1.5^b^	8.26 ± 0.8^b^	3.011±0.7^b^
A+F	4.90 ± 0.1^d^	7.45 ± 0.2^d^	5.65 ± 0.4^d^	4.91 ± 0.4^d^	1.662±0.1^d^
C-A+F	4.86 ± 0.2^d^	7.17 ± 0.3^d^	5.43 ± 0.6^d^	4.16± 0.4^d^	0.539±0.4^e^

*IC_50_ (μM TE/mg): the concentration at which 50% activity is inhibited

**DW, distilled water extract; A, alcalase hydrolysis; C-A, condensed alcalase hydrolysis; F, flavourzyme hydrolysis; C-F, condensed flavourzyme hydrolysis; A+F, alcalase+flavourzyme hydrolysis; C-A+F, condensed alcalase+flavourzyme hydrolysis

***Each value is expressed as mean ± standard deviation (n = 3)

Values not sharing a common letter in a row are significantly different at *p* < 0.05 by Dunnett’s multiple range tests.

## Discussion

Proteins contain AAs, which are important elements of food nutrition. Protein quality depends on the types of AAs present. Xiaoming et al (2010) evaluated the protein contents of 100 species from a number of insect orders [30]. Here, we determined the crude protein contents from samples and found significant variation among our samples. The crude protein contents ranged from 4.01% to 39.41% (Table 1). C-A+F (39.41%) and C-A (34.45%) showed higher protein contents than the other samples. Consistent with these findings, previous studies have shown that alcalase resulted in the highest protein recovery compared with other proteolytic enzymes [31,32].

Here, significant variations in FAAs contents among the different samples were observed. DW extracted a lower amount of total FAAs compared with enzymatic extraction. A extracted lower total FAAs compared with F (Table 3). This result is supported by a previous report in which the combination of both alcalase and flavourzyme yielded increased FAAs contents as compared with alcalase or flavourzyme alone [12]. They also reported that hydrolysis with flavourzyme or the combination of flavourzyme and alcalase both produced significant amounts of FAAs. We also found that F and C-F produced more FAAs than A and C-A. We speculated that it may be due to the different characteristics of enzyme.

Among the AAs examined in this study, all essential AAs tended to be predominant in A and C-A; in particular, C-A+F yielded the highest levels of free Leu. Interestingly, DW extraction was unable to extract Cys and Ser. Moreover, a negligible amount of Asn was extracted from DW, whereas flavourzyme effectively extracted Asn. Asn is known to be essential for brain development [33]. Furthermore, Park (1995) reported that Asn inhibits the toxicity of alcohol [34]. Additionally, the amounts of essential AAs, including Leu, Tyr, Lys, Ala, and Val, were higher than the amounts of other AAs.

All enzymatic samples were rich in hydrolyzed Glu, which is often used as a food additive and flavor enhancer [35](Table 4).

Alcalase is used to extract hydrophobic AAs, such as Ala, Val, Leu, Iso, Pro, Phe, Try, Cys, and Met, whereas flavourzyme is effective for extraction of hydrophilic amino acids, including Ser, Thr, Asn, Glu, His, and Tyr [36,37]. Consistent with these previous results, we found that F yielded more Ser, Thr, Asn, Gln, and Tyr as hydrophilic FAAs than A. Moreover, A+F was more effective for both hydrophobic and hydrophilic AAs extraction, compared with A or F, respectively. Among the hydrophilic AAs, Val was the most abundant, whereas Leu was the most abundant among the hydrophobic AAs in A+F (Table 3). In addition, protein hydrolysates produced by alcalase often have a bitter taste owing to their high content of hydrophobic AAs [36,37]. However, flavourzyme may reduce bitterness. Therefore, effective extraction can be achieved using the combination of alcalase and flavourzyme [37].

Free radicals are highly reactive species produced in the body during normal metabolic functions or introduced from the environment. Free radicals contribute to a wide range of conditions and diseases in humans, including arthritis, ischemia and reperfusion injury of many tissues, central nervous system injury, gastritis, and cancer. Therefore, discovery of natural antioxidants derived from food sources and food processing by-products has attracted much attention. The body possesses defense systems, including enzymes and antioxidant nutrients, which alleviate the damaging effects of reactive oxygen species. However, imbalances between free radical production and the antioxidant defense system may responsible for oxidative stress. The antioxidant activities of *T*. *molitor* larvae may not be attributed to a single mechanism. therefore, five methods were used to evaluate the antioxidant activities from different aspects. Therefore, we next evaluated the antioxidant activity of samples by performing DPPH, hydroxyl radical, hydrogen peroxide scavenging, FRAP, and ORAC assays. DPPH, FRAP, and ORAC assays are widely used to determine antioxidant activity [28,38,39]. Hydroxyl radicals are highly reactive and can react instantly with food and biologically related substrates [38]. Hydrogen peroxide is not a free radical; however, due to its extreme reactivity, it is responsible for the production of reactive oxygen species. The ORAC assay is most applicable because it uses a biologically relevant radical source [40]. All samples scavenged free radicals in a concentration-dependent manner (Fig 2) The IC_50_ values of ORAC, FRAP, and DPPH, hydroxyl radical, and hydrogen peroxide scavenging activities are shown in Table 5. DW showed the lowest antioxidant activity of all the samples, whereas A+F and C-A+F exhibited the highest antioxidant activity. Moreover, A and C-A showed higher scavenging activities than F and C-F. However, there were no significant differences in antioxidant activities between A and C-A or between F and C-F (Table 5, Fig 2). These results were positively correlated with the HAAs composition. We observed similar inhibitory activities for both A+F and C-A+F when used at a concentration of 5 mg/mL. A+F showed the highest levels of DPPH, hydroxyl free radical, and hydrogen peroxide scavenging activities at 83, 38, and 73 μM TE/mg, respectively, whereas C-A+F showed scavenging activities of 83, 40, and 73 μM TE/mg, respectively (Fig 2A–2C). Additionally, C-A+F showed higher FRAP and ORAC activities than A+F when used at a concentration of 5 mg/mL (Fig 2D and 2E). In our results, the C-A+F of the highest protein content and antioxidant capacity. Elias et al., think that “Proteins can inhibit lipid oxidation by biologically designed mechanisms (e.g. antioxidant enzymes and iron-binding proteins) or by nonspecific mechanisms. Both of these types of antioxidative proteins contribute to the endogenous antioxidant capacity of foods [41]. This is consistent with the direction of our results.

Taken together, these results indicated that all enzymatic extracts were a good source of antioxidants, with C-A+F producing the best results.

## Supporting information

S1 Fig Various antioxidant activities of *Tenebrio molitor* larvae. A,1,1-Diphenyl-2-picryl-hydrazyl (DPPH) radical scavenging activity.
(XLSX)

S2 Fig Various antioxidant activities of *Tenebrio molitor* larvae. B, hydroxyl radial scavenging activity.
(XLSX)

S3 Fig Various antioxidant activities of *Tenebrio molitor* larvae. C, hydrogen peroxide scavenging activities.
(XLSX)

S4 Fig Various antioxidant activities of *Tenebrio molitor* larvae. D, oxygen radical absorbance capacity (ORAC).
(XLSX)

S5 Fig Various antioxidant activities of *Tenebrio molitor* larvae. E, ferric reducing antioxidant power (FRAP).
(XLSX)
